# Impact of Electron Acceptor Availability on Methane-Influenced Microorganisms in an Enrichment Culture Obtained From a Stratified Lake

**DOI:** 10.3389/fmicb.2020.00715

**Published:** 2020-05-14

**Authors:** Sigrid van Grinsven, Jaap S. Sinninghe Damsté, John Harrison, Laura Villanueva

**Affiliations:** ^1^Department of Marine Microbiology and Biogeochemistry, NIOZ Royal Netherlands Institute for Sea Research, Utrecht University, Utrecht, Netherlands; ^2^Department of Earth Sciences, Faculty of Geosciences, Utrecht University, Utrecht, Netherlands; ^3^School of the Environment, Washington State University Vancouver, Vancouver, WA, United States

**Keywords:** methanotroph culture, nitrate, electron acceptor, *Methylobacter*, microaerobic, methane oxidation, oxygen concentration

## Abstract

Methanotrophs are of major importance in limiting methane emissions from lakes. They are known to preferably inhabit the oxycline of stratified water columns, often assumed due to an intolerance to atmospheric oxygen concentrations, but little is known on the response of methanotrophs to different oxygen concentrations as well as their preference for different electron acceptors. In this study, we enriched a methanotroph of the *Methylobacter* genus from the oxycline and the anoxic water column of a stratified lake, which was also present in the oxic water column in the winter. We tested the response of this *Methylobacter*-dominated enrichment culture to different electron acceptors, i.e., oxygen, nitrate, sulfate, and humic substances, and found that, in contrast to earlier results with water column incubations, oxygen was the preferred electron acceptor, leading to methane oxidation rates of 45–72 pmol cell^−1^ day^−1^. Despite the general assumption of methanotrophs preferring microaerobic conditions, methane oxidation was most efficient under high oxygen concentrations (>600 μM). Low (<30 μM) oxygen concentrations still supported methane oxidation, but no methane oxidation was observed with trace oxygen concentrations (<9 μM) or under anoxic conditions. Remarkably, the presence of nitrate stimulated methane oxidation rates under oxic conditions, raising the methane oxidation rates by 50% when compared to oxic incubations with ammonium. Under anoxic conditions, no net methane consumption was observed; however, methanotroph abundances were two to three times higher in incubations with nitrate and sulfate compared to anoxic incubations with ammonium as the nitrogen source. Metagenomic sequencing revealed the absence of a complete denitrification pathway in the dominant methanotroph *Methylobacter*, but the most abundant methylotroph *Methylotenera* seemed capable of denitrification, which can possibly play a role in the enhanced methane oxidation rates under nitrate-rich conditions.

## Introduction

Methane is the second most important greenhouse gas on earth, and a direct reduction in methane emissions is needed to keep global temperatures below the goal of 1.5°C above pre-industrial levels (Rogelj et al., 2018). Methanotrophy, the microbial conversion of methane to carbon dioxide, is a key process in limiting methane emissions from aquatic systems. Segarra et al. (2015) estimated the decrease in freshwater wetland emissions by methane oxidation to be up to 50%, while Martinez-Cruz et al. (2018) estimated that up to 34% of produced methane in lake sediments is consumed by methanotrophy. In marine systems, anaerobic oxidation of methane (AOM) is estimated to reduce methane emissions by 90% (Knittel and Boetius, 2009). A consortium of anaerobic methane-oxidizing archaea (ANME) and sulfate-reducing bacteria using sulfate as the electron acceptor for methane oxidation is responsible for this process (Boetius et al., 2000). In the water column of freshwater systems, these archaea are rarely detected, likely due to their zero tolerance to oxygen. Many anoxic lakes and reservoirs experience regular or irregular intrusions of oxygen, which make these systems less suitable habitats for ANME. Methane-oxidizing bacteria (MOB) are often detected in freshwater systems, at the oxic–anoxic interface and, more rarely, in the anoxic water column (e.g., Rudd and Hamilton, 1975; Harrits and Hanson, 1980; Biderre-Petit et al., 2011; Blees et al., 2014; Milucka et al., 2015; Oswald et al., 2016; Michaud et al., 2017). Although most methanotrophs require oxygen to oxidize methane, MOB are often assumed to prefer low-oxygen conditions over oxygen saturation. Several studies suggest an inhibitory effect of atmospheric oxygen concentrations on the methane oxidation rate (Rudd and Hamilton, 1975; Van Bodegom et al., 2001; Danilova et al., 2016; Thottathil et al., 2019). A few species of MOB have been described that could potentially use electron acceptors other than oxygen, such as nitrite (Ettwig et al., 2010) and nitrate (Kits et al., 2015; Oswald et al., 2017; Rissanen et al., 2018). Sulfate has also been suggested as an electron acceptor in freshwater sediments, but not in the water column (Schubert et al., 2011). Organic matter and humic substances, which are shown to be able to function as both an electron donor and acceptor (Lovley et al., 1996; Klüpfel et al., 2014; Valenzuela et al., 2019), have been suggested to play a role in AOM in lakes (Saxton et al., 2016; Reed et al., 2017), but have so far only been shown to impact aquatic AOM performed by ANME in marine (Scheller et al., 2016) and tropical wetland systems (Valenzuela et al., 2017, 2019).

Several studies (Murase and Frenzel, 2007; Jones and Grey, 2011; Sanseverino et al., 2012) have shown that methane-derived carbon is an important contributor to aquatic food webs on different scales. Many microbes cannot use methane and therefore depend on the conversion of methane-derived carbon by methanotrophs. Generally, methane-derived carbon is assumed to end up in methanotroph biomass or CO_2_, the main reaction product of methane oxidation. However, under oxygen-limited conditions, MOB have been shown to excrete metabolites such as methanol, formaldehyde, formate, acetate, and succinate (Xin et al., 2004, 2007; Kalyuzhnaya et al., 2013; Gilman et al., 2017), which can be used by other members of the microbial community.

This study aims to expand the knowledge of how oxygen and other potential terminal electron acceptors affect methanotrophs, especially *Methylobacter*, which occur naturally in oxic, microoxic, and anoxic zones of stratified lake water columns. Previously, we showed that *Methylobacter* sp. is an important methanotroph in the seasonally stratified Lake Lacamas, and water column incubation experiments revealed that it is capable of methane oxidation under a variety of conditions (van Grinsven et al., 2019). Here, we describe the establishment of an enrichment culture dominated by *Methylobacter* and used it to evaluate the effects of the concentration of the potential electron acceptor (oxygen, nitrate, sulfate, and humic substances) on the methane oxidation rates and microbial community structure using 16S ribosomal RNA (rRNA) gene amplicon sequencing. Furthermore, the metabolic potential of selected microbial groups stimulated in the enrichment cultures was also determined by a metagenomic sequencing approach.

## Experimental Setup

### Sample Collection

Suspended particulate matter samples were collected on 9 April 2018 from the center of Lacamas Lake, WA, USA (45.62N, 122.43W). Lacamas Lake is a seasonally stratified, hypereutrophic system with an average depth of 7.8 m and maximum depth of 19.8 m, which is on the Environmental Protection Agency list of impaired and threatened waters. It is monomictic, with stratification occurring yearly in May and a turnover mixing period from October to December. During sampling, the lake was not stratified, as determined using a Hydrolab DS5X sonde (Hach, Loveland, USA) with sensors for conductivity, temperature, dissolved oxygen, and pH. At the moment of sampling, the oxygen concentration was >350 μM throughout the water column, the temperature 4–8°C, and the methane concentration <1 μM. Water was collected from 12 m depth using a VanDorn sampler, stored in carboys, and transported back to the lab, where it was filtered within 96 h over 47 mm 0.7 μm pore size glass fiber filters. Filters were stored in non-filtered lake water from 12 m depth and kept at 4°C until shipment and further processing.

### Cultivation

The suspended particulate matter that was collected on the filters was scraped off and transferred under oxic conditions to 20 ml nitrate mineral salts (NMS) medium (Whittenbury et al., 1970) in a 120 ml acid-washed and autoclaved glass pressure bottle with butyl rubber stopper. A flow scheme is shown in Figure S1. Methane (1 ml, 99.99% pure) was added and the bottle was stored at 15°C in the dark. Every 2 weeks, the pressure bottle was opened under oxic conditions, and 2 ml of the cell-containing medium was transferred to 18 ml fresh sterile NMS medium in a sterile 120 ml glass pressure bottle with butyl stopper, after which 1 ml methane was added again. These steps were repeated every 2 weeks. After 8 weeks, the resulting enrichment culture was studied using catalyzed reporter deposition fluorescence *in situ* hybridization (CARD-FISH) with probes MLB482 (targeting *Methylobacter*; Gulledge et al., 2001) and Creno445 (targeting *Crenothrix*; Oswald et al., 2017), following the protocol as described on https://www.arb-silva.de/fish-probes/fish-protocols. The medium was filtered over a 10 μm mesh glass fiber filter (Whatmann) to separate cell clusters from single cells, as illustrated in Figure S1. The cell material that remained on the filter was scraped off and transferred to a sterile 120 ml bottle with NMS media. The steps described above were repeated for this enrichment culture. The amount of biomass was increased by replicating the subculture in eight 500 to 1,000 ml glass bottles. After 8 weeks, the cells were harvested by centrifugation at 2,800 × *g* for 5 min. The supernatant was discarded and all biomass of the enrichment cultures was combined to create one uniform concentrated enrichment culture in NMS medium. Cell density was not measured. A 20 ml aliquot was used for DNA analysis.

### Incubation Experiments With the Enrichment Culture

Two sets of incubation experiments were performed using the methanotroph enrichment culture. The first set of experiments was aimed at the response of *Methylobacter* to the electron acceptors nitrate (in the presence and absence of oxygen), sulfate, and humic substances and is referred to as the “electron acceptor experiments.” The second set of experiments, referred to as the “O_2_ concentration experiment,” was set up to study the response of *Methylobacter* sp. to different oxygen concentrations. An overview of the experimental setup of these two experiments is provided in Table S1.

All experiments were performed in triplicate. “Electron acceptor experiments” were performed in 260 ml acid-washed and autoclaved glass bottles with butyl rubber stoppers, with a total volume of 210 ml media. The O_2_ concentration experiments were performed in 120 ml bottles containing 70 ml media. The media of the “anoxic incubation” bottles and all bottles of the O_2_ concentration experiments were prepared using boiled ultrapure water to minimize the initial oxygen concentration of the media. Each incubation bottle was inoculated with the same amount of concentrated enrichment culture. All media in the anoxic bottles was bubbled with nitrogen for 20 min to remove residual oxygen, after which the bottles were closed, crimp sealed, and the headspace was flushed and exchanged with N_2_ gas using a GRInstruments (Wijk bij Duurstede, the Netherlands) automatic gas exchanger. Abiotic controls were set up identically to the bottles for the anoxic experiments, but were not inoculated with the concentrated enrichment culture. This resulted in a lower liquid volume and, therefore, in a methane concentration ±120 μM lower than that in the anoxic incubations.

All bottles were supplemented with 2.6 ml 100% methane (Sigma-Aldrich), shaken vigorously for 1 min to establish equilibrium between the gas and the water phase, and the methane concentration in the gas phase was subsequently measured by gas chromatography with flame ionization detection (GC-FID; Thermo Scientific Focus GC). The bottles were subsequently incubated at 15°C in the dark. Bottles were shaken at sampling moments.

#### Electron Acceptor Incubation Experiments

“Electron acceptor incubations” lasted 3 days for the oxic experiments and 33 days for the anoxic incubation experiments. Incubation experiments with nitrate (i.e., oxic and anoxic nitrate incubations) were performed with the same NMS medium that was used for cultivation, as described above, containing nitrate as the only nitrogen source (Whittenbury et al., 1970). Control, sulfate-supplemented, and humic-supplemented incubations of the electron acceptor experiments were performed with an AMS medium, containing ammonium rather than nitrate as the nitrogen source (1 g L^−1^ KNO_3_ was replaced with 0.5 g L^−1^ NH_4_Cl, as described by Whittenbury et al., 1970). As the enrichment culture used for inoculation was in the NMS media, relatively small amounts of nitrate were introduced into the control, sulfate-supplemented, and humic-supplemented incubation experiments. Anoxic nitrate-supplemented bottles of the electron acceptor experiments were amended with 0.3 g additional KNO_3_ (in addition to the KNO_3_ that was present in the NMS media). To the sulfate-supplemented bottles, 0.35 g Na_2_SO_4_ was added (target concentration, 0.012 M). The humic substance-supplemented bottles contained 1 g of commercially available humic acids mixture (Sigma-Aldrich). Methane concentrations in the headspace were measured by extracting 50 μl gas using a gas-tight syringe, daily during the first 4 days and irregularly after this initial phase. All methane analyses using a GC-FID were performed in triplicate. Methane oxidation rates were determined using linear regression analysis (Microsoft Excel version 16.16.10).

Upon termination of the experiment, all bottles were sampled for DNA by filtering the contents of the individual bottles over individual 47 mm 0.2 μm pore size polycarbonate filters. All samples were stored at −80°C until DNA was extracted by using the RNeasy Powersoil Total RNA extraction + DNA elution kits. DNA extracts were kept at −80°C until further processing.

#### O_2_ Concentration Experiments

All experiments were performed with the NMS medium and the same concentrated culture used to inoculate the electron acceptor experiments, although 3 weeks were in between the start of the electron acceptor experiments and O_2_ concentration experiments. All bottles of the O_2_ concentration experiments were set up as anoxic bottles and left for 2 days after setup, after which the bottles were randomly divided into four groups, of which three received air injections. Bottles for the anoxic experiment received no injection, “trace oxygen” bottles received 20 μl air ([O_2_] 7.5–9 μM), “microoxic” bottles received 160 μl air ([O_2_] 23–30 μM), and “saturated oxygen” bottles received 5,000 μl air ([O_2_] ±600 μM). The methane concentration in all bottles was measured on days 3 and 5, after which the “saturated oxygen” incubations were terminated. The “microoxic” and “trace oxygen” bottles received another air injection on days 6 and 13, identical to the volume of the first injections. On day 14, all incubations were terminated. DNA was sampled following the same procedure as described above, but extraction was done with the RNeasy Powersoil DNA extraction kit, after which the DNA extracts were kept at −80°C until further processing.

### 16S rRNA Gene Analysis

The general 16S rRNA archaeal and bacteria primer pair 515F and 806RB targeting the V4 region (Caporaso et al., 2012) was used for the 16S rRNA gene amplicon sequencing and analysis, as described in Besseling et al. (2018), with a melting temperature of 56°C. PCR products were gel purified using the QIAquick Gel-Purification kit (Qiagen), pooled, and diluted. Sequencing was performed by the Utrecht Sequencing Facility (Utrecht, the Netherlands) using an Illumina MiSeq sequencing platform (Caporaso et al., 2010). The 16S rRNA gene amplicon sequences were analyzed by the Cascabel pipeline (Asbun et al., 2019), including quality assessment by FastQC (Andrews, 2010), assembly of the paired-end reads with Pear (Zhang et al., 2014), library demultiplexing, operational taxonomic unit (OTU) clustering, and representative sequence selection (“longest” method) by diverse Qiime scripts (Caporaso et al., 2010). The OTU clustering algorithm was uclust (Edgar, 2010) with an identity threshold of 97% and assign taxonomy with BLAST (Altschul et al., 1990) by using the Silva 128 release as the reference database (https://www.arb-silva.de/; Quast et al., 2013). To compare the *Methylobacter* OTUs, we focused on OTUs with relative abundances >0.4% of the total 16S rRNA gene reads.

16S rRNA gene copies were quantified using quantitative PCR (qPCR) with the same primer pairs as used for amplicon sequencing (515F, 806RB). The qPCR reaction mixture (25 μl) contained 1 U of Pico Maxx high-fidelity DNA polymerase (Stratagene, Agilent Technologies, Santa Clara, CA), 2.5 μl of 10 × Pico Maxx PCR buffer, 2.5 μl of 2.5 mM of each dNTP, 0.5 μl bovine serum albumin (20 mg ml^−1^), 0.02 pmol μl^−1^ of primers, 10,000 times diluted SYBR Green® (Invitrogen) (optimized concentration), 0.5 μl of MgCl_2_ (50 mM), and ultrapure sterile water. The cycling conditions for the qPCR reaction were the following: initial denaturation at 98°C for 30 s, 45 cycles of 98°C for 10 s, and 56°C for 20 s, followed by a plate read, 72°C for 30 s, and 80°C for 25 s. Specificity of the reaction was tested with a gradient melting temperature assay from 55 to 95°C, with 0.5°C increments of 5 s. The qPCR reactions were performed in triplicate with standard curves encompassing a range from 10^3^ to 10^7^ molecules μl^−1^. qPCR efficiency for the 16S rRNA gene quantification was 103.7%, with *R*^2^ = 0.980. For quantification of the microbial groups, we make the simplifying assumption that all microorganisms of the microbial community in Lacamas Lake contained a single 16S rRNA gene copy in their genome.

Representative sequences were extracted from the dataset and compared with closely related sequences by performing a phylogenetic analysis using the maximum likelihood method and the General Time-Reversible model in MEGA6 (Tamura et al., 2013). Additionally, the phylogenetic placement of the metagenome-assembled genome (MAG) LL-enrich-bin26 (Table S2), attributed to the *Methylobacter* genus, was further assessed and compared to the MAG bin63 of the *Methylobacter* clade 2 reported in van Grinsven et al. (2019) by using Phylosift (v. 1.0.1) (Darling et al., 2014) based on 34 marker genes, as described in van Grinsven et al. (2019). The 16S rRNA amplicon reads (raw data) have been deposited in the NCBI Sequence Read Archive (SRA) under BioProject number PRJNA598329, BioSamples SAMN13712582–SAMN13712612.

### Metagenome Analysis

The sample that was selected for metagenomic sequencing originated from the 10 μm filtrate, (Figure S1). DNA was extracted as described above and used to prepare a TruSeq DNA nano-library, which was further sequenced with Illumina MiSeq 2 × 300 bp, generating over 46 million 2 × 300-bp paired-end reads. Data was analyzed with an in-house pipeline as described in van Grinsven et al. (2019). The binning of MAGs was performed with DAS Tool with penalty for duplicate marker genes and a megabin penalty of 0.3. Quality of the MAGs was assessed using CheckM v1.0.7 running the lineage-specific workflow (Parks et al., 2015). MAGs were annotated with Prokka v1.12 (Seemann, 2014) and by the Rapid Annotation using Subsystem Technology (RAST) pipeline v2.0 (Aziz et al., 2008). The annotation of key metabolic pathways was refined manually. In order to classify the MAGs according to their relative abundance in the sequenced sample, MetaBAT was run again by using the abundance estimation (total average depth, average abundance, or also called average coverage of each contig included in the bin) generated by MetaSPAdes and checked again with CheckM, as included in Table S2. The completeness and redundancy of the MAG bins was assessed by the DAS_Tool Package (Sieber et al., 2018). The taxonomic classification of the MAGs of interest was determined by using GTDB-Tk (v0.3.2; http://gtdb.ecogenomic.org) (Table S2). The metagenome of the sample specified in Table S3 is available in NCBI under BioProject number PRJNA598329, BioSample SAMN13712974. The sequence raw data of the MAGs LL-enrich-bin26 and bin28 are deposited in NCBI under BioSample numbers SAMN13735002 and SAMN13735003, respectively.

## Results

The most abundant methanotroph of Lacamas Lake, a seasonally stratified lake, is a *Methylobacter* species; it was detected in the oxic water column in the winter and in the microoxic oxycline and the anoxic hypolimnion in the summer (van Grinsven et al., 2019). In order to be able to further study the response of this methanotroph to different concentrations of oxygen and other electron acceptors, an enrichment culture was established.

### Enrichment Culture Microbial Community

**Figure 1:**
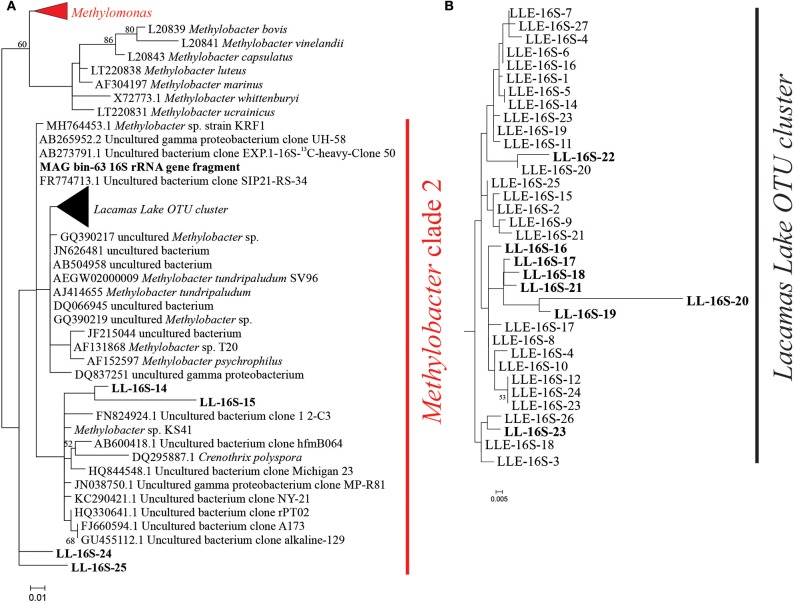
**(A)** Phylogenetic 16S rRNA gene tree in which the representative sequences of the methanotrophic groups detected in the 16S rRNA gene amplicon sequencing analysis (i.e., *Methylomonas* and *Methylobacter*) of the incubation experiments are indicated in *red*. **(B)** Zoom in on the Lake Lacamas *Methylobacter* cluster as defined in the text. *LL-16S-number* sequences in *bold* represent operational taxonomic unit (OTU) sequences previously detected in the Lacamas Lake water column and the water column incubations as described in van Grinsven et al. (2019). *LLE-16S-number* sequences correspond to the *Methylobacter* OTU sequences detected in this study and are listed in Table S4. The *MAG bin63* 16S rRNA gene sequence corresponds to the 16S rRNA sequence of the most abundant MAG bin in a water column incubation experiment sample which was taxonomically assigned to *Methylobacter*, as described in van Grinsven et al. (2019). The phylogenetic analysis was restricted to the sequence fragment (~290 bp) obtained with the 16S rRNA amplicon sequencing analysis. Maximum likelihood estimation was performed using the General Time-Reversible model.

The enrichment culture was dominated by gene sequences attributed to *Methylobacter* clade 2 (43%; Figure 1) (Smith et al., 2018), accompanied by 2.8% of *Methylomonas* sp. and 0.1% other methanotrophs, all part of the order Methylococcales (Table 1). The *Methylobacter* OTUs with the highest relative abundances were LLE-16S-2, LLE-16S-7, LLE-16S-8, LLE-16S-10, and LLE-16S-12 (Table S4). These OTUs form a phylogenetic subcluster of closely related sequences (i.e., 96–99% similarity; Supplementary File 1) in the *Methylobacter* clade 2 cluster (i.e., the Lacamas Lake OTU cluster; Figure 1B) together with the detected sequences in the Lacamas Lake water column (i.e., LL-16S-number). The most closely related cultured species was *Methylobacter tundripaludum* (Figure 1A).

**Table 1:** Relative abundance of 16S rRNA gene reads (% of total) attributed to methylotrophs and 16S rRNA copies per liter in the sample as determined using quantitative PCR.

	**Sample**		**Electron acceptor experiment**						**O**_****2****_ **concentration experiment**			
	**Starting lake water**	***Methylobacter* sp. enrichment culture**	**Control, oxic**	**Nitrate, oxic**	**Control, anoxic**	**Nitrate, anoxic**	**Sulfate, anoxic**	**Humics, anoxic**	**Saturated**	**Microoxic**	**Trace**	**Anoxic**
*Methylobacter* spp. (%)	0.6	43	44	38	11	25	19	1.6	23	21	19	20
*Methylomonas* spp. (%)	0.2	2.8	4.6	6.6	0.4	4.8	1.2	0.3	1.4	1.9	2	1.9
Other Methylococcales (%)	0.02	0.1	0.5	0.5	0.3	0.5	0.4	0.2	0.2	0.3	0.3	0.2
*Methylotenera* spp. (%)	1	21	15	22	13	17	14	14	12	11	13	11
Total 16S rRNA copies per liter	n.d.	n.d.	1.8 × 10^7^	1.5 × 10^7^	2.5 × 10^7^	1.8 × 10^7^	1.8 × 10^7^	1.3 × 10^7^	1.9 × 10^7^	2.1 × 10^7^	1.5 × 10^7^	1.6 × 10^7^
Methanotroph cells per liter^a^	n.d.	n.d.	4.3 × 10^6^	3.3 × 10^6^	1.5 × 10^6^	2.6 × 10^6^	1.9 × 10^6^	0.1 × 10^6^	2.3 × 10^6^	2.4 × 10^6^	1.6 × 10^6^	1.7 × 10^6^

*n.d., not determined*.

a *Calculated with assuming two copies of the 16S rRNA gene per Methylobacter cell, three copies per Methylomonas cell, and one copy per “other Methylococcales” cell*.

**Figure 2:**
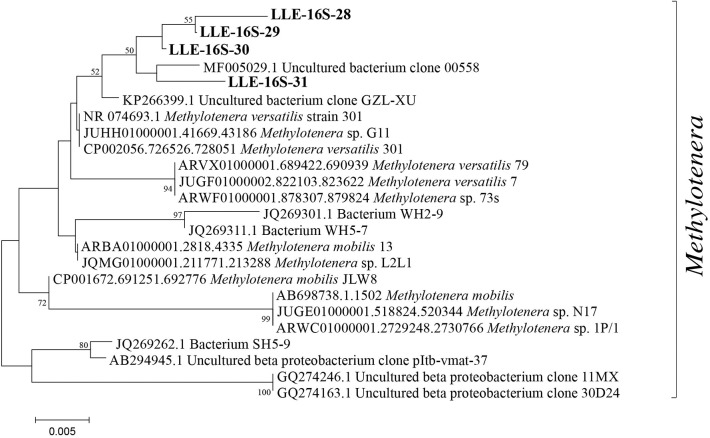
Phylogenetic 16S rRNA gene tree with representative sequences of the operational taxonomic units (OTUs) classified as *Methylotenera*, indicated in *bold*. The phylogenetic analysis was restricted to the sequence fragment (~290 bp) obtained with the 16S rRNA amplicon sequencing analysis. Maximum likelihood estimation was performed using the General Time-Reversible model.

Apart from *Methylobacter* sp., also bacteria of the genus *Methylotenera* were highly abundant in the enrichment culture. They represent 21% of the total 16S rRNA gene copies (Table 1). The detected OTUs classified as *Methylotenera* clustered with two uncultured bacterium clones; the most closely related cultured species was *Methylotenera versatilis* (Figure 2). Bacteria of the genus *Flavobacterium* were also relatively abundant in the enrichment culture (5.5%; Table 2), as well as members of the order Burkholderiales (8.3%; Table 2).

**Table 2:** Relative abundance of 16S rRNA gene reads (% of total) of other microbial groups discussed in the manuscript.

	**Sample**		**Electron acceptor experiment**						**O**_****2****_ **concentration experiment**			
	**Starting lake water**	**Enrichment culture**	**Control, oxic**	**Nitrate, oxic**	**Control, anoxic**	**Nitrate, anoxic**	**Sulfate, anoxic**	**Humics, anoxic**	**Saturated**	**Microoxic**	**Trace**	**Anoxic**
*Brevundimonas*	0.1	0.5	0.6	0.5	15	3.2	10	1.3	2.2	3.2	3.1	3.5
Burkholderiaceae	15	8.3	12	6	11	11	10	30	9.5	10	10	10
*Flavobacterium*	1.5	5.5	4	5.4	18	10	16	16	12	14	16	14
*Pseudomonas*	0.2	0.1	0.3	0.2	3	2	1.3	5.9	0.5	1.1	1	0.9
Rhodocyclaceae	0.8	0.2	0.4	0.4	1.9	0.9	1.9	2.1	0.9	1.8	1.8	1.8
*Sulfuritalea*	0.4	0.2	0.2	0.2	1.0^a^	0.5	1.3	0.9	0.2	0.5	0.5	0.6

a *High standard deviation between triplicate incubations of 0.45%*.

### Metabolic Potential of the Main Microbial Components of the Enrichment Culture

In order to characterize the metabolic potential of the main microbial components of the enrichment culture, we performed metagenomic sequencing of a sample derived from the 10 μm filtrate (see Figure S1). *Methylobacter* sp. was less abundant than in the enrichment (i.e., 22 vs. 43% the total 16S rRNA gene reads). However, the distribution of the OTUs attributed to *Methylobacter* spp. in this sequenced sample was similar to that reported in the enrichment culture (Table S4). High relative abundances of *Methylotenera* (i.e., 24%) and *Methylomonas* (17%) were also evident (Table S3).

**Figure 3:**
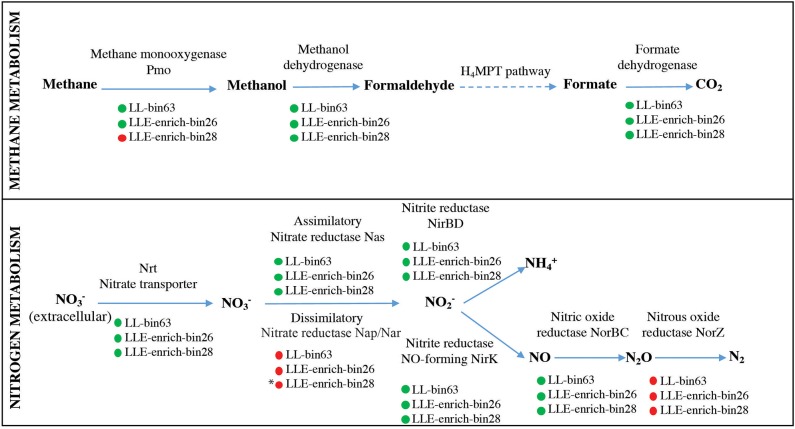
Description of the genes present in the *Methylobacter* LLE-enrich-bin26 and *Methylotenera* LLE-enrich-bin28 regarding their methane and nitrogen metabolic pathways and comparison with the *Methylobacter* MAG LL-bin63 previously obtained from incubations with Lacamas Lake water samples (van Grinsven et al., 2019). *Green* and *red circles* indicate the presence/absence of the coding gene. * indicates that *Methylotenera* LLE-enrich-bin28 may have the potential to perform dissimilatory nitrate reduction in the absence of the *Nap/Nar* gene, as explained in the text.

Metagenome sequencing resulted in three most abundant MAG bins affiliated to the methanotrophs *Methylobacter* sp. (i.e., LLE-enrich-bin26), *Methylomonas* sp. (i.e., LLE-enrich-bin27), and to the methylotroph *Methylotenera* sp. (i.e., LLE-enrich-bin28) (Table S2). Here, we focus on the metabolic characterization of the MAG bins affiliated to *Methylobacter* and *Methylotenera* due to their higher relative abundances in the enrichment culture (Table 1), specifically of the genetic potential of the nitrogen and methane and carbon metabolism. The MAG LLE-enrich-bin26 is taxonomically classified as a *Methylobacter* sp. and harbors all the genes encoding for the particulate methane monooxygenase (pMMO; see Supplementary File 2), allowing for the conversion from methane to methanol, while the *Methylotenera* MAG LLE-enrich-bin28 lacks this gene (Supplementary File 3; Figure 3). The genes required for the further conversion from methanol to CO_2_ are present in both MAGs (see Figure 3). Regarding the nitrogen metabolism pathways, both the *Methylobacter* and *Methylotenera* LLE-enrich-bin26 and bin28 MAGs harbor the genes encoding for nitrate transporters, assimilatory nitrate reductase (Nas), nitrite reductase (NirBD) to ammonia and to nitric oxide (NirK), as well as the gene coding for the nitric oxide reductase (NorBC) to nitrous oxide (N_2_O), but not the genes coding for the nitrous oxide reductase (NorZ) to dinitrogen gas (Figure 3).

### Microbial Community Composition and Methane Consumption in Incubation Experiments With Different Electron Acceptors

**Figure 4:**
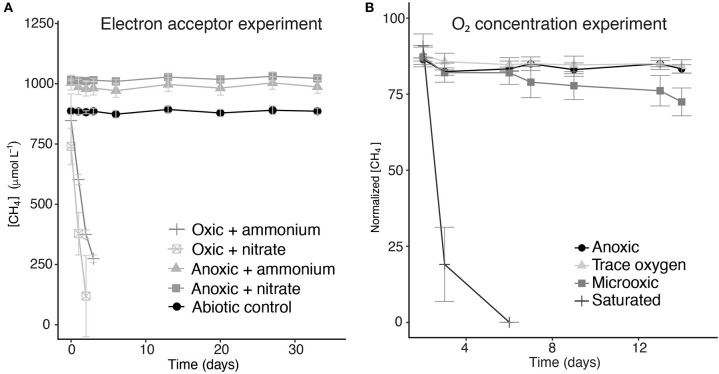
Methane concentrations over time during the incubation experiments with different electron acceptors **(A)** and normalized methane concentrations in incubation experiments with different oxygen concentrations **(B)**. *Error bars* represent the standard error of triplicate incubations. The methane concentrations over time of the incubations to which sulfate and humic substances were added are not shown, but were very similar to the ammonium- and nitrate-supplemented anoxic incubations.

Two sets of incubation experiments were performed using the methanotroph enrichment culture obtained. The first set of experiments was aimed at the response of *Methylobacter* sp. to the electron acceptors nitrate (in the presence and absence of oxygen), sulfate, and humic substances. In all oxic experiments, methane was consumed rapidly (Figure 4). The experiments were terminated within 2–3 days in anticipation of methane depletion. The net methane consumption rate of the incubation with nitrate was higher than that in the control incubation with ammonium (310 and 200 μmol L^−1^ day^−1^, respectively). We estimated that the total number of methanotrophic bacteria in the oxic incubations was 3.3 × 10^6^–4.3 × 10^6^ cells L^−1^ (Table 1).

The methane turnover rate per cell is, therefore, estimated to be 45 and 72 pmol cell^−1^ day^−1^ for the control and nitrate-amended oxic incubations, respectively. No net methane consumption could be detected under anoxic conditions, even with the addition of the alternative electron acceptors nitrate, sulfate, or humic substances (Figure 4). Nitrate concentration measurements showed no clear difference between the nitrate concentrations at the start and the end of the experiment (Figure S2), mainly due to large variations between the samples and the high starting concentrations.

The relative abundance of *Methylobacter* sp. was significantly higher (*p* < 0.05; Table S5) in the two oxic incubations of the electron acceptor experiment when compared to the anoxic incubations. The *Methylobacter* abundance in the oxic incubations (43 and 38% for the control and nitrate-supplemented, respectively; Table 1) was not significantly different due to substantial variations between replicates. The addition of different electron acceptors in the anoxic incubations changed the microbial community (Tables 1, **3**). The addition of nitrate or sulfate resulted into a significantly (*p* < 0.05; Table S5) higher *Methylobacter* abundance (25 and 18%, respectively; Table 1) compared to the anoxic control (11%; Table 1). *Methylobacter* OTUs LLE-16S-2 and LLE-16S-7 were the most abundant in the oxic incubations, similarly to the enrichment culture (Table S4). LLE-16S-12, which was highly abundant in the enrichment culture, became less dominant in the incubations. Similar to the oxic incubations, LLE-16S-2 and LLE-16S-7 were the most abundant *Methylobacter* OTUs in the anoxic control and nitrate incubations, with, in addition, a relatively high abundance of LLE-16S-9 (Table S4). The sequences closely related to *Methylotenera* (Figure 2) remained relatively abundant in all incubation experiments (14–29%; Table 1). Bacteria of the genera *Flavobacterium Brevundimonas* and *Pseudomonas* had higher relative abundances in the anoxic than in oxic incubations, both with and without nitrate, although *Brevundimonas* was more abundant in the anoxic incubations without nitrate (Table 2). *Brevundimonas* comprised 15 and 3% of the total microbial abundance in the anoxic control and nitrate-supplemented incubations, respectively. The genus *Sulfuritalea* was more abundant in the anoxic sulfate incubations (1.3%) than in nitrate incubations (0.5%; Table 2). The microbial community composition of the incubation with added humic substances was completely different compared to the other anoxic incubations (Tables 1, 2), with remarkably high relative abundances of bacteria of the order Burkholderiales and the family Comamonadaccea (31 and 16%, respectively). The relative abundance of total archaeal 16S rRNA gene sequences was below 0.5% in all incubations.

### Microbial Community Composition and Methane Consumption in Incubation Experiments With Different Oxygen Concentrations

The second set of incubation experiments performed with the *Methylobacter* sp. enrichment culture was aimed at the response of *Methylobacter* sp. to different oxygen conditions. We incubated the enrichment culture under saturated ([O_2_] >600 μM), microoxic ([O_2_] 23–30 μM), trace oxygen ([O_2_] 7.5–9 μM), and anoxic conditions.

The methane consumption rates were two orders of magnitude higher under oxygen saturation condition than under microoxic conditions (520 and 6.4 μM day^−1^, respectively). Under trace oxygen and anoxic conditions, no methane consumption was observed (Figure 4B). Based on the measured concentrations of methane and the estimated concentrations of oxygen in the vials, a ratio of methane and oxygen consumption was calculated. The oxygen concentration in the saturated oxygen incubations (±640 μM) was, assuming methanotrophy was the only process consuming oxygen, present in surplus and, thus, sufficient for a 2:1 molar ratio of oxygen/methane usage. In the microoxic incubation bottles, between 6 and 8 μmol of methane was consumed over the whole duration of the experiment. The amount of oxygen present in the microoxic incubations was estimated based on oxygen measurements and injected air volume, to be maximum 7.1 μmol, allowing for a maximum ratio of 1:1 in the oxygen/methane usage.

The relative abundance of the 16S rRNA gene sequences attributed to *Methylobacter* was highest in the incubation under saturated oxygen conditions (23%; Table 1), but the absolute abundances of all methanotrophs (including *Methylotenera, Methylomonas, Methylotenera*, or other Methylococcales) were not significantly different between the oxic, microoxic, trace, and anoxic experiments (1.6–2.3 × 10^6^ cells L^−1^; Table 1). Overall, the communities of the microoxic, suboxic, and anoxic incubations were similar, whereas the community under saturated oxygen conditions was significantly different, with lower relative abundances of all non-methanotrophic species, as listed in Table 2.

## Discussion

Lacamas Lake, the source of the material used for our enrichment culture, contained uncultured *Methylobacter* species thriving in the oxic and anoxic water columns as well as in the microoxic oxycline (van Grinsven et al., 2019). Incubations with water column samples revealed that these bacteria oxidized large amounts of methane (72 μM day^−1^) under anoxic conditions in the stratified summer water column, stimulated by the addition of both nitrate and sulfate (van Grinsven et al., 2019), but were also naturally present in the oxic, methane-depleted winter water column. Phylogenetic analysis showed that the *Methylobacter* species of the Lacamas Lake summer and winter water columns and incubations grouped closely together with the *Methylobacter* species that dominated the enrichment culture (i.e., 96–99% similarity; Figure 1). *Methylobacter* and related methanotrophs have been previously detected in lakes, mostly under microoxic conditions ([O_2_] ±60 μM) (Rudd and Hamilton, 1975; Harrits and Hanson, 1980; Oswald et al., 2016; Michaud et al., 2017), but also in anoxic environments, such as sediments or anoxic lake waters (Biderre-Petit et al., 2011; Milucka et al., 2015; Martinez-Cruz et al., 2017). Although most bacteria falling in the *Methylobacter* group are known as aerobic methanotrophs, it has recently been suggested that specific species contain the genomic potential to perform anaerobic methane oxidation, or methane oxidation under strong oxygen limitation, by coupling methane oxidation to nitrate reduction (Svenning et al., 2011; Smith et al., 2018) or by using a fermentation pathway (van Grinsven et al., 2019). Knowledge on the effect of other electron acceptors (i.e., sulfate and humic substances) on *Methylobacter* sp. is, however, lacking, and often the biochemical pathways involved in methanotrophy under anoxic conditions remain unclear (Biderre-Petit et al., 2011; Blees et al., 2014; Martinez-Cruz et al., 2017; Reed et al., 2017). Despite the increase in methane oxidation rates (from 9 to 72 μM day^−1^) that was observed in anoxic Lacamas Lake incubations with the addition of nitrate, the genome of the dominant *Methylobacter* species did not encode all the genes required to perform denitrification, and its mechanisms for anaerobic methane oxidation therefore remain unclear (van Grinsven et al., 2019).

In the current study, we aimed to determine the preference of the *Methylobacter* species, and other methanotrophs present in Lacamas Lake, for oxygen concentrations and electron acceptors other than oxygen, such as nitrate, by means of laboratory incubations with an enrichment culture.

### *Methylobacter* sp. in Water Column and Enrichment Culture Incubations

The *Methylobacter* OTU sequences detected in the enrichment culture obtained from Lacamas Lake were closely related to the sequences previously detected both in Lacamas Lake water column and incubation studies with lake water samples, as was confirmed by the 16S rRNA gene phylogeny (Figure 1). In addition, the *Methylobacter* MAG bin obtained from the enrichment culture (i.e., LLE-enrich-bin26) was also closely related to the *Methylobacter* MAG previously obtained in an incubation with Lacamas Lake water (i.e., LL-bin63) (van Grinsven et al., 2019; see Figure S3). Therefore, we conclude that the *Methylobacter* species obtained in the enrichment culture in this study are representative of those existing in Lacamas Lake and can thus be used to draw conclusions on their electron acceptor and oxygen preferences, which can be extrapolated to the conditions in the original system. Both the two *Methylobacter* MAGs coincided in their genetic potential to oxidize methane, perform mixed-acid fermentation from pyruvate to succinate and H_2_ (Figure S4), as well as in harboring an incomplete denitrification pathway (Figure 3). Several methanotrophs contain parts of the denitrification pathway, but only few species have been shown to couple methane oxidation to denitrification (Smith et al., 2018). Based on its genetic potential, the *Methylobacter* species present in our incubation experiments could be capable of dissimilatory nitrite reduction, but as no nitrite was provided in the incubation experiments, we do not expect this pathway to be relevant for methane oxidation.

### *Methylotenera–Methylobacter* Co-occurrence

Bacteria of the genus *Methylotenera*, which were highly abundant in our enrichment culture incubations (11–22%), have often been detected in co-occurrence with methanotrophs and have been shown to use reaction products of methanotrophy (Yu and Chistoserdova, 2017), coupling methanol oxidation to nitrate reduction (Kalyuzhnaya et al., 2011). Their relative abundances increased not only in the enrichment culture but also in water column incubations with high methane oxidation rates (van Grinsven et al., 2019); an interaction between *Methylobacter* and *Methylotenera* species is, therefore, not unlikely. The *Methylotenera* MAG LLE-enrich-bin28 has the genomic potential to oxidize methanol (Figure 3), but lacks the *pmoA* gene necessary for the oxidation of methane. Its denitrification pathway seems incomplete as the gene encoding for the dissimilatory reduction of nitrate to nitrite (*Nap*/*Nar* gene) was missing. A mutant phenotype study on *Methylotenera mobilis*, however, demonstrated that the single subunit nitrate reductase (*Nap*), Mmol_1648, appears to be involved in both the assimilatory and dissimilatory denitrification pathways (Mustakhimov et al., 2013). The nitrate reductase (*Nas*) detected in our *Methylotenera* MAG LLE-enrich-bin28 was homologous to the nitrate reductase (*Nap*) of *M. mobilis*. The *Methylotenera* species detected in our incubations may therefore also be able to perform denitrification, similarly to the *Methylotenera* species that have been described in the literature before (*M. mobilis* and *M. versatilis*: Lapidus et al., 2011; Mustakhimov et al., 2013).

### Role of Nitrate and Ammonium in Methane Oxidation

The methane oxidation rates of the oxic incubation experiments were higher than those observed previously in environmental studies (Eller et al., 2005; Schubert et al., 2010; Blees et al., 2014), but a proper comparison between an enrichment culture and environmental studies is difficult to make. The methane oxidation rates in the oxic incubations with nitrate were significantly higher than those in the ammonium control incubations (311 and 195 μmol L^−1^ day^−1^, respectively), despite the fact that the methanotroph abundance was higher in the oxic control (8.6 × 10^6^ copies L^−1^ in the control and 6.7 × 10^6^ copies L^−1^ in the nitrate-amended incubations). Ammonium (${\text{NH}}_{4}^{+}$), which was added to the control experiment as the nitrogen source, can lower the methanotrophic activity due to the structural similarity between CH_4_ and ${\text{NH}}_{4}^{+}$, causing competitive inhibition (Bédard and Knowles, 1989). The affinity of the methane monooxygenase enzyme for CH_4_ is, however, 600- to 1,300-fold higher than the affinity for ${\text{NH}}_{4}^{+}$, so we expect this effect to be of little influence. Generally, ammonium stimulates methanotroph growth and protein synthesis by providing bioavailable nitrogen (Bodelier et al., 2000), although recent research in soils found a decrease in methane oxidation rates after ammonium addition (Walkiewicz et al., 2018). Nitrate has also been suggested in earlier studies to inhibit methane oxidation under oxic conditions (Geng et al., 2017; Walkiewicz and Brzezinska, 2019), although the observed effect in those studies could have been due to the high salt concentrations, not specifically nitrate (Dunfield and Knowles, 1995), or due to the conversion of nitrate to nitrite (Roco et al., 2016), which is known to be an inhibitor of methane oxidation (Dunfield and Knowles, 1995; Hutsch, 1998).

As we consider ammonium inhibition unlikely, we assume a stimulating effect of nitrate on the oxic methane oxidation rate. As discussed above, the dominant *Methylobacter* species in both the enrichment cultures as well as the water column lack the genes for a complete denitrification pathway. A complete assimilatory nitrate reduction pathway was present, and nitrate can thus be used for protein synthesis, enhancing growth. Another possibility would, however, be an interaction with *Methylotenera*, which is likely capable of denitrification. *Methylotenera* could function as a syntrophic partner for *Methylobacter*, as has been observed in several methane-oxidizing bacteria and archaea (Boetius et al., 2000; Milucka et al., 2015; Krause et al., 2017). Whether such a partnership indeed exists in our incubation experiments requires more research.

### *Methylobacter* sp. Under Oxygen Limitation

Surprisingly, in contrast to the water column incubation studies, in which methane oxidation by *Methylobacter* was the highest under oxygen-limiting conditions (van Grinsven et al., 2019), methane oxidation in incubations with the enrichment culture was the highest under oxygen-saturated conditions (Figure 4B). Methane oxidation under low-oxygen conditions (microoxic; O_2_, 23–30 μM) occurred, but was much less efficient than the methanotrophy under oxygen saturation conditions. The oxygen concentration in the closed bottles was measured only at the start of the incubations, and the concentrations may thus have changed over the course of the experiments. Air was, however, injected into the microoxic and trace oxygen incubation bottles on days 2, 6, and 13 in order to prevent oxygen depletion. Despite being aerobes, methanotrophs are generally assumed to be (partially) inhibited by oxygen concentrations >60 μM or at least stimulated by low-oxygen conditions (Rudd and Hamilton, 1975; Van Bodegom et al., 2001; Danilova et al., 2016; Walkiewicz et al., 2018; Thottathil et al., 2019; Walkiewicz and Brzezinska, 2019), resulting in a low methane oxidation efficiency at high oxygen concentrations. A recent study by Thottathil et al. (2019) stated that methane oxidation rates are only at 20% of their maximum value at oxygen saturation and that the fact that this oxygen inhibition is generally not considered for global models may offset the total methane oxidation potential calculations greatly, expressing the need for additional studies on the response of methanotrophs to different oxygen concentrations. Our data reveal that this general assumption about the oxygen inhibition of methanotrophy is not correct for the *Methylobacter* species present in this lake system.

The methane oxidation detected in the microoxic conditions may depend partially on a fermentative pathway, as was also suggested for methanotrophs in the Lacamas Lake water column (van Grinsven et al., 2019), with an energy yield too low for cell growth but supporting only cell maintenance. It, however, remains unclear why the *Methylobacter* cells in the trace oxygen and anoxic incubations, which possibly went into a dormant state, remain almost as abundant as the *Methylobacter* cells in the oxic and microoxic experiments, while no methane oxidation and, thus, no energy production seemed to take place in the first two. Similarly, methanotrophs remained a substantial part of the community in the anoxic electron acceptor incubations despite no detectable methane oxidation, with higher *Methylobacter* abundances in the nitrate- and sulfate-amended incubations compared to the control (19 and 25%, 4.3 × 10^6^ and 3.3 × 10^6^ methanotroph cells per liter in the nitrate and sulfate incubations, respectively, while only 11%, 2.6 × 10^6^ methanotroph cells per liter in the anoxic control). The DNA method used cannot distinguish between dead, dormant, or active cells, but the strong contrast between the nitrate and sulfate incubations, and the incubations with humic substances, in which a major reduction in *Methylobacter* relative abundance to 1.6% and a decrease in methanotroph abundance to 1 × 10^5^ cells L^−1^ (Table 1) was observed, suggests that a difference between the treatments exists. Methanotrophs were shown to have an efficient survival mechanism under starvation in anoxic conditions compared to starvation under oxic conditions (Roslev and King, 1995), increasing their chance of survival under stress conditions.

Methane oxidation occurred directly after oxygen injection into the oxic and microoxic bottles (Figure 4), despite the fact that the cultures were under anoxic conditions for several days before the start of the experiment. It is unknown whether the cells were in a dormant state under anoxic conditions, but these results showed that no recovery time was needed, therefore implying a fast adaptation mechanism. This ability to rapidly adapt to anoxic or oxic conditions could be a strategy of methanotrophs living in dynamic environments, such as seasonally stratified water columns, allowing them to rapidly adapt to the changing conditions of their niche.

Fermentation-based methane oxidation, which could potentially be performed by *Methylobacter* under trace oxygen conditions, has been shown to occur under extremely low methane oxidation and growth rates (1.75 nmol min^−1^ mg^−1^ protein) (Kalyuzhnaya et al., 2013). Rates like these were below the detection limit of our methods, opening the possibility of low-rate methane oxidation in the trace oxygen incubations.

### Methane Oxidation Under Anoxic Conditions

No methane oxidation was observed under the anoxic conditions in the *Methylobacter* enrichment culture obtained in this study despite *Methylobacter* being present and active under the anoxic conditions in the incubations performed with the water column samples (van Grinsven et al., 2019). Possibly, the anaerobic methane oxidation rates were too low to detect by our methods. Rates in anoxic lake waters have been reported to be in the range of 0.1–2.5 μM day^−1^ (Blees et al., 2014; Oswald et al., 2016). If comparable rates would occur in our anoxic incubations, the result would be a total decrease in methane of 3.2–80 μM over the full 32-day period, which would be difficult to detect given the large fluctuations between our measurements. The measured methane oxidation rates in the Lacamas Lake anoxic water column were, however, much higher (up to 45 μM day^−1^) (van Grinsven et al., 2019). Simultaneous methane production, counteracting the decrease in the concentration of methane caused by oxidation, could also have masked methane consumption. Methane production in anoxic systems is commonly observed, both in environmental and culture studies (Reeburgh, 2007; Conrad et al., 2011; Grasset et al., 2018), and could be fueled by the reaction products of methane oxidation by *Methylobacter*, such as acetate or methanol (Oremland and Polcin, 1982). We did, however, not detect commonly known methane producers such as methanogenic archaea with the 16S rRNA gene diversity analysis.

Possibly, non-methanotrophic members of the microbial community, which are present in the natural community of the Lacamas Lake water column, are essential in mediating methane oxidation under anoxic conditions. These microbes may not have been selected in the oxic enrichment process used in this study. In this regard, Oswald et al. (2015) showed that methanotrophs in the anoxic hypolimnion of Lake Rotsee were dependent on phototrophic microorganisms for the production of oxygen to mediate their methane oxidation pathway. This pathway was not relevant in our incubations, which were performed in the dark, but a similar collaboration between a non-methanotrophic species and *Methylobacter* species may be essential in mediating methane oxidation under anoxic conditions. A possible candidate could be bacteria of the genus *Sulfuritalea*, which were abundant in the water column incubations in which anoxic methane oxidation was observed (van Grinsven et al., 2019), but which were only present in low relative abundance in the enrichment culture and the incubation experiments of the current study (Table 2). They could be potentially involved as a partner in anoxic methane oxidation due to their capabilities of nitrate reduction (Kojima and Fukui, 2011). In contrast, bacteria of the order Burkholderiales were abundant in both the water column incubations and the enrichment culture incubations, although they were most abundant in the enrichment incubations with humic substances, which actually contained the lowest abundance of methanotrophs (Tables 1, 2). Another possibility could be the composition of the medium. The enrichment culture incubation experiments were performed on a rich media, including common trace metals and a vitamin solution. Certain compounds may, however, have been present in the lake water, which were missing in the medium. Lanthanides, part of the rare earth elements, have been shown to affect *Methylobacter* (Krause et al., 2017) and were not added to the enrichment medium. Possibly, compounds like these were lacking in the enrichment incubation experiments and limited anaerobic methane oxidation.

## Conclusions

Studies have found methanotrophs at a wide range of locations and environmental conditions. Despite these observations, little is known about the drivers of the spatial distribution that is observed, while recent research stressed the importance of a correct representation of the nonlinear response of methane oxidation rates to oxygen concentrations (Thottathil et al., 2019). The effect of nitrogen and oxygen concentrations on methanotrophs was shown to differ strongly between similar environments, likely due to the different organic carbon contents (Walkiewicz and Brzezinska, 2019), indicating that the relationships between the methane oxidation rates, methanotroph abundance, nitrogen source, and oxygen concentration are complicated and that more work is needed to understand these relationships. Our study shows that *Methylobacter* sp., a methanotroph often assumed to thrive under low-oxygen conditions, preferred high-oxygen conditions over a microoxic environment under laboratory conditions. When comparing this data with an environmental study with the same *Methylobacter* species, we, however, saw that the oxygen response of this species is dependent on factors we do not yet fully understand, potentially involving interactions with other non-methanotrophic microorganisms present in the same system. More research is therefore needed to reveal the pathways and microorganisms involved in the aerobic and anaerobic methane oxidation by this *Methylobacter* species.

## Data Availability Statement

The 16S rRNA amplicon reads (raw data) have been deposited in the NCBI Sequence Read Archive (SRA) under BioProject number PRJNA598329, BioSamples SAMN13712582-SAMN13712612. The metagenome of the sample specified in Table S3 is available in NCBI under BioProject number PRJNA598329, BioSample SAMN13712974. The sequence raw data of the MAGs LL-enrich-bin-26, and bin-28 are deposited in NCBI under BioSample numbers SAMN13735002 and SAMN13735003, respectively.

## Author Contributions

SG designed and conducted the experiments under the supervision of LV and JS. LV and JH assisted in designing the experiment. SG and LV analyzed the data. SG wrote a first draft of the manuscript, to which all authors contributed in subsequent revisions.

## Conflict of Interest

The authors declare that the research was conducted in the absence of any commercial or financial relationships that could be construed as a potential conflict of interest.

## Supplementary Material

Figure S1 Overview of experimental methods. The lake graphic represents Lacamas Lake, with on the left side the mixed winter water column, homogeneously low in methane and rich in oxygen, and on the right side the stratified summer water column, with a methane-rich oxygen-depleted deeper water layer and a methane-poor and oxygen-rich top water layer. Samples for this study were collected in the mixed winter water column, transferred onto nitrate mineral salts (NMS) media **(A)** and received methane. The enrichment culture was subcultured six times, by transferring culture to fresh media rich in methane in a 1:20 dilution, before using size separation **(B)** to increase the *Methylobacter* relative abundance. The particulate matter that remained on the 10 μm filter was scraped off and suspended in fresh media **(C)**. The resulting cultures were again subcultured six times **(D)**, and afterwards combined and concentrated to create one concentrated culture that was used to set up incubation experiments **(E)**. A sample for metagenomic sequencing was obtained via subculturing of the filtrate **(F)**. This metagenomic sequencing sample is described in Table S3. For more details, see the Experimental setup section.

Figure S2 Nitrate concentration at the start and end of the electron acceptor incubation experiments. Error bars represent the standard error over triplicate incubations.

Figure S3 Maximum likelihood phylogenetic tree based on 34 concatenated single-copy, protein-coding genes (following the method of Dombrowski et al., 2018) of the MAG bin LLE-enrich-bin26 and the MAGs as described in van Grinsven et al. (2019) (i.e., bin-63, bin-37, and bin-19).

Figure S4 Predicted pathway for mixed-acid fermentation from pyruvate to succinate and H2 production in the *Methylobacter* LL-bin63 and *Methylobacter* LLE-enrich-bin26 (Green and red circles indicate presence/absence of the coding gene).

Table S1 Overview of the media (AMS - ammonium mineral salts, NMS - nitrate mineral salts) used in the incubation experiments, and of the additions of methane, additional nitrate, sulfate or humic substances to the incubation experiments.

Table S2 Characteristics of the most abundant MAGs detected in the sample derived from the 10 μm filtrate (Figure S1), which contained a high diversity in *methanotrophs* (i.e. 22% *Methylobacter* and 17% *Methylomonas*) and a high relative abundance of *Methylotenera* (i.e. 24%) based on 16S rRNA gene amplicon sequencing. Avg, average. Classification was inferred by GTDB-Tk as indicated in the material and methods.

Table S3 Composition of the sample that was used for metagenome sequencing.

Table S4 Relative abundance (%) of *Methylobacter* OTUs (>0.4% in at least one of the samples) in respect to the total 16S rRNA gene reads in the amplicon sequencing analysis for each incubation and for the enrichment sample used for the metagenomic sequencing.

Table S5 P-value of t-tests between *Methylobacter* relative abundances of the three replicates per incubation type, indicating whether the difference in relative abundance between sample categories was statistically significant.

Supplementary File 1 Rapid Annotation using Subsystem Technology (RAST) annotation of the MAG LLE-enrich-26 Methylobacter.

Supplementary File 2 Rapid Annotation using Subsystem Technology (RAST) annotation of the MAG LLE-bin28 Methylotenera.

Supplementary File 3 Percentage of similarity of the sequences included in the phylogenetic tree of Figure 2.
