# Supplementary figures and images for: Impact of Electron Acceptor Availability on Methane-Influenced Microorganisms in an Enrichment Culture Obtained From a Stratified Lake
Source: Front Microbiol. 2020 May 14;11:715. doi: 10.3389/fmicb.2020.00715 (PMC7240106; doi:10.3389/fmicb.2020.00715)

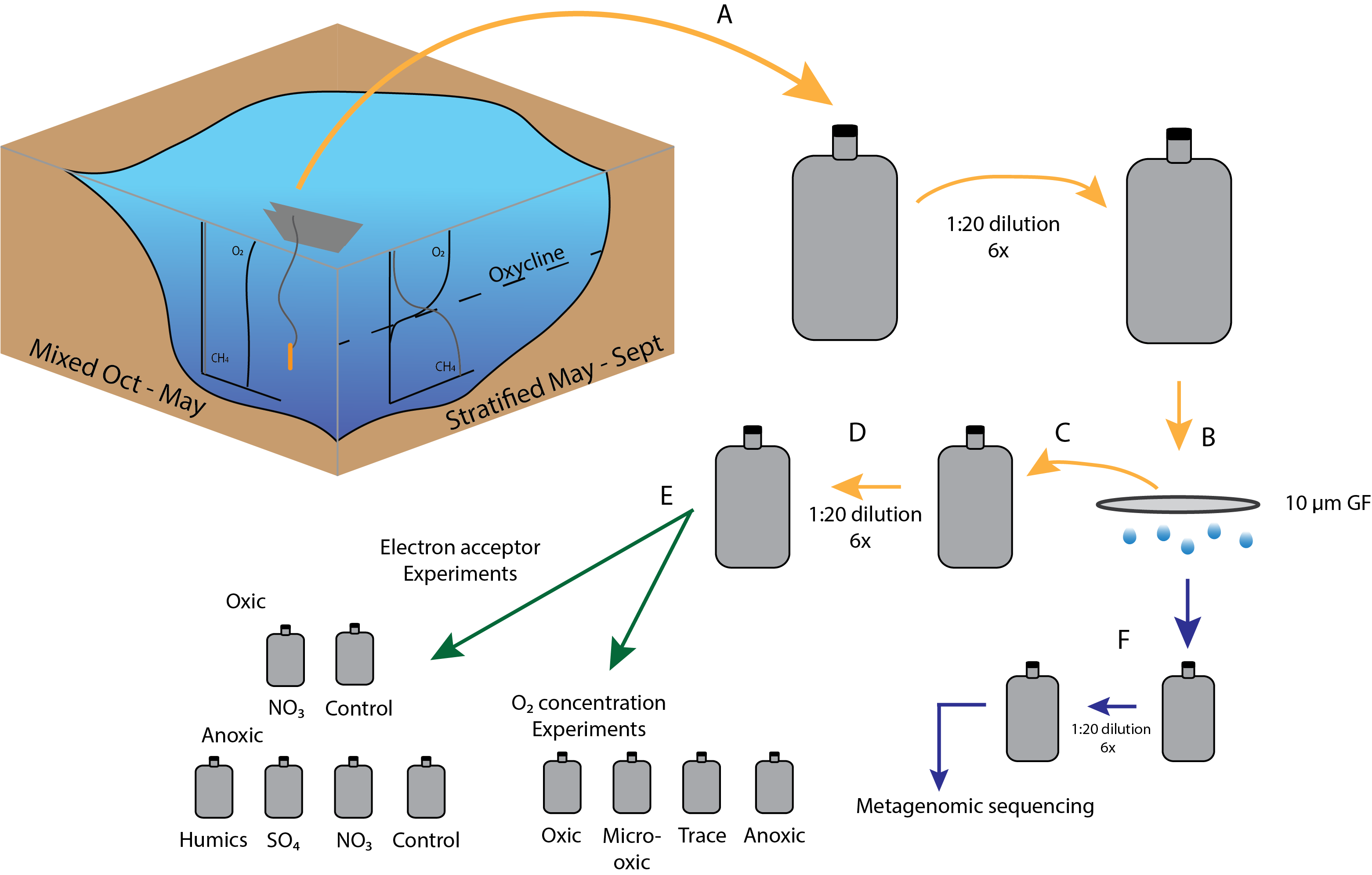

Supplement: Figure S1 — Overview of experimental methods. The lake graphic represents Lacamas Lake, with on the left side the mixed winter water column, homogeneously low in methane and rich in oxygen, and on the right side the stratified summer water column, with a methane-rich oxygen-depleted deeper water layer and a methane-poor and oxygen-rich top water layer. Samples for this study were collected in the mixed winter water column, transferred onto nitrate mineral salts (NMS) media (A) and received methane. The enrichment culture was subcultured six times, by transferring culture to fresh media rich in methane in a 1:20 dilution, before using size separation (B) to increase the Methylobacter relative abundance. The particulate matter that remained on the 10 μm filter was scraped off and suspended in fresh media (C). The resulting cultures were again subcultured six times (D), and afterwards combined and concentrated to create one concentrated culture that was used to set up incubation experiments (E). A sample for metagenomic sequencing was obtained via subculturing of the filtrate (F). This metagenomic sequencing sample is described in Table S3. For more details, see the Experimental setup section. [file Image_1.PNG]

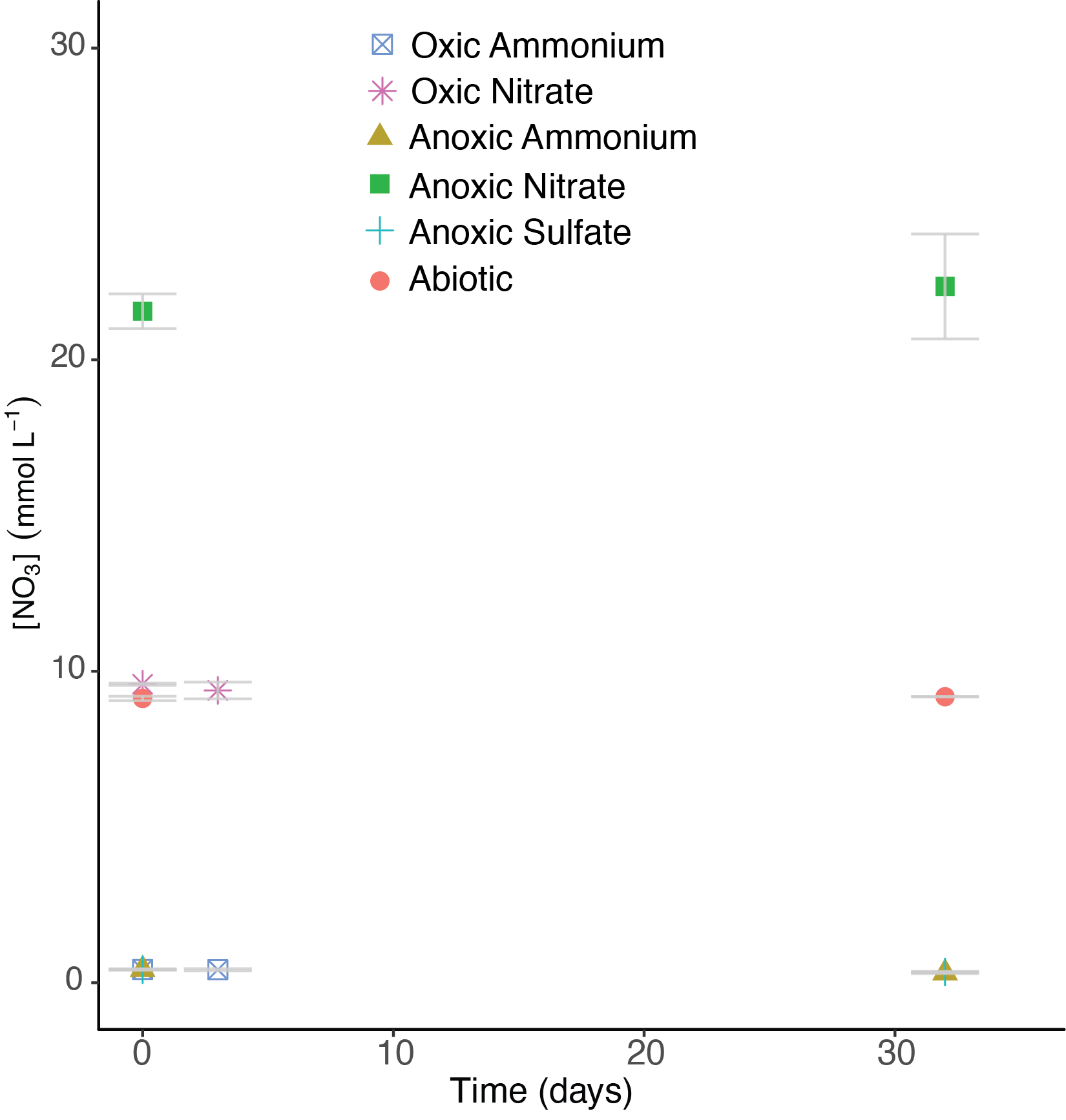

Supplement: Figure S2 — Nitrate concentration at the start and end of the electron acceptor incubation experiments. Error bars represent the standard error over triplicate incubations. [file Image_2.PNG]

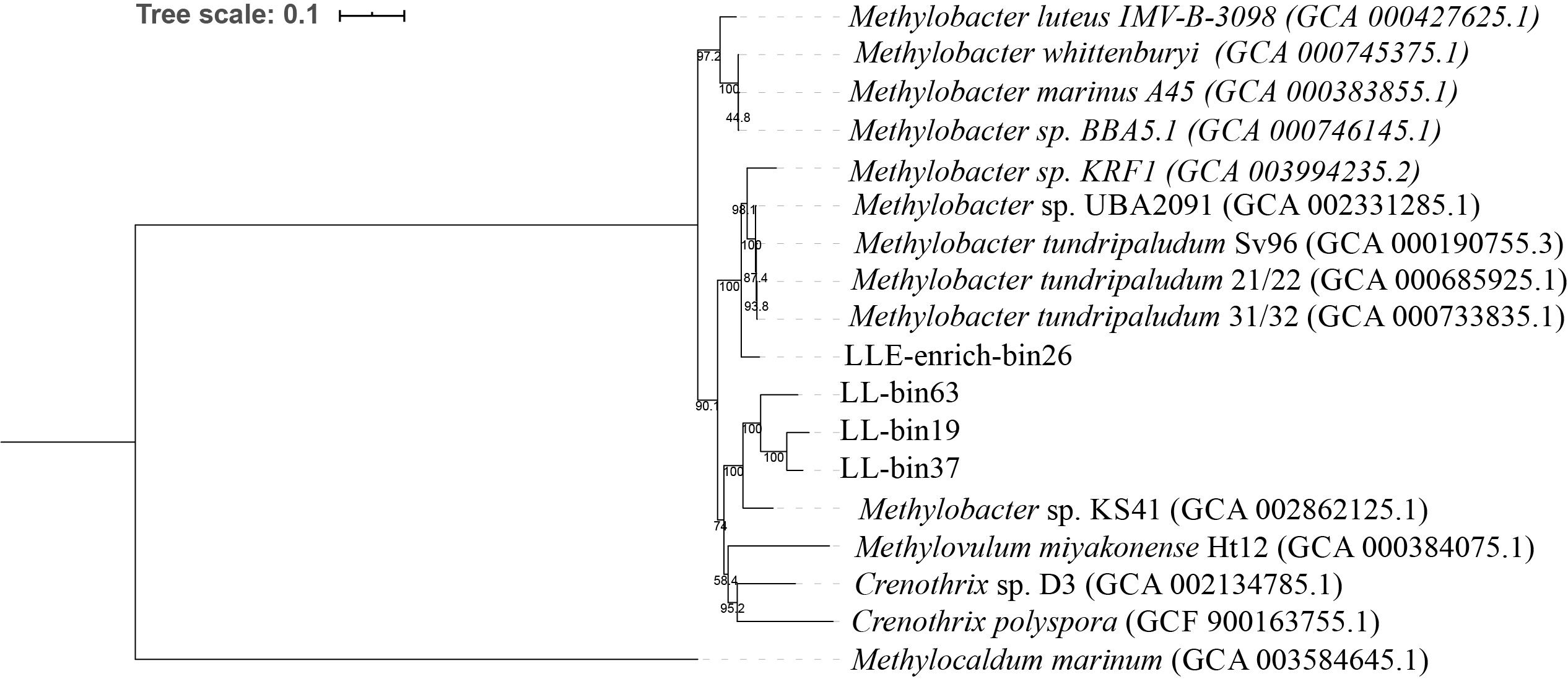

Supplement: Figure S3 — Maximum likelihood phylogenetic tree based on 34 concatenated single-copy, protein-coding genes (following the method of Dombrowski et al., 2018) of the MAG bin LLE-enrich-bin26 and the MAGs as described in van Grinsven et al. (2019) (i.e., bin-63, bin-37, and bin-19). [file Image_3.JPEG]

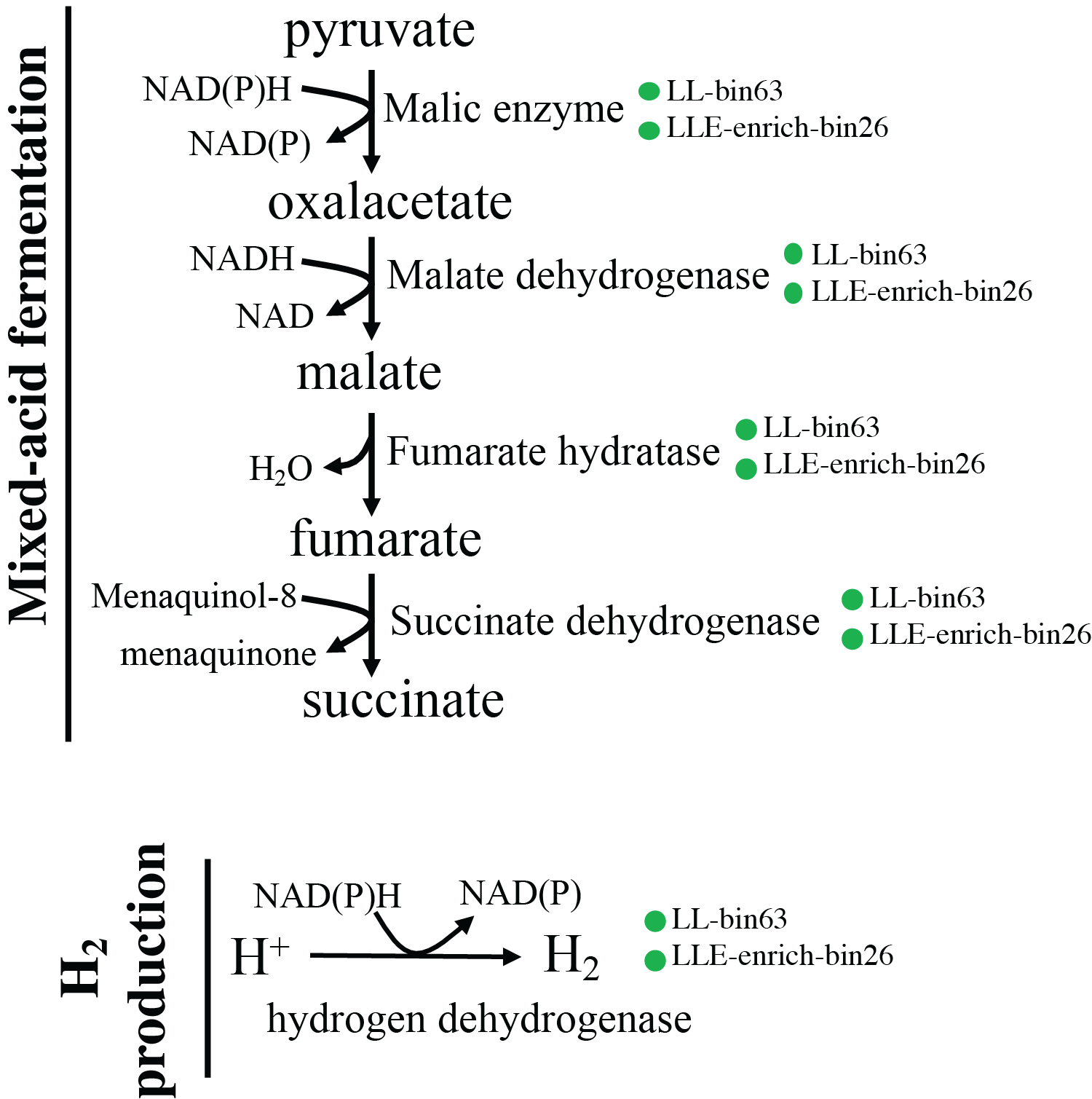

Supplement: Figure S4 — Predicted pathway for mixed-acid fermentation from pyruvate to succinate and H2 production in the Methylobacter LL-bin63 and Methylobacter LLE-enrich-bin26 (Green and red circles indicate presence/absence of the coding gene). [file Image_4.PNG]
