# Supplementary material for: Impact of Electron Acceptor Availability on Methane-Influenced Microorganisms in an Enrichment Culture Obtained From a Stratified Lake
Source: Front Microbiol. 2020 May 14;11:715. doi: 10.3389/fmicb.2020.00715 (PMC7240106; doi:10.3389/fmicb.2020.00715)
Supplement: Table S1 — Overview of the media (AMS - ammonium mineral salts, NMS - nitrate mineral salts) used in the incubation experiments, and of the additions of methane, additional nitrate, sulfate or humic substances to the incubation experiments. [file Data_Sheet_4.docx]

**Table S1.** Overview of the media (AMS – ammonium mineral salts, NMS – nitrate mineral salts) used in the incubation experiments, and of the additions of methane, additional nitrate, sulfate or humic substances to the incubation experiments.

|  | “Electron acceptor experiment” | | | | “O_2_-concentration experiment” | | | | |
| --- | --- | --- | --- | --- | --- | --- | --- | --- | --- |
|  | Control | Nitrate | Sulfate | Humics | Saturated | Micro-oxic | Trace | Anoxic |  |
| AMS media | x |  | x | x |  |  |  |  |  |
| NMS media |  | x |  |  | x | x | x | x |  |
| CH_4_ | x | x | x | x | x | x | x | x |  |
| Additional NO_3_^-^ |  | x |  |  |  |  |  |  |  |
| Na_2_SO_4_ |  |  | x |  |  |  |  |  |  |
| Humic substances |  |  |  | x |  |  |  |  |  |

**Table S2.** Characteristics of the most abundant MAGs detected in the sample derived from the 10 μm filtrate (Fig. S1), which contained a high diversity in methanotrophs (i.e. 22% *Methylobacter* and 17% *Methylomonas*) and a high relative abundance of *Methylotenera* (i.e. 24%) based on 16S rRNA gene amplicon sequencing. Avg = average. Classification was inferred by GTDB-Tk as indicated in the material and methods.

| Bin | Contigs | Bases | Avg Abundance | Completeness | Redundancy | GTDBTK Tax |
| --- | --- | --- | --- | --- | --- | --- |
| LL-enrich-bin26 | 415 | 4213939 | 615.934 | 96.08 | 35.29 | d__Bacteria;p__Proteobacteria;c__Gammaproteobacteria;o__Methylococcales;  f__Methylomonadaceae;g__Methylobacter_A;s__ |
| LL-enrich-bin27 | 436 | 4488068 | 583.699 | 66.67 | 13.73 | d__Bacteria;p__Proteobacteria;c__Gammaproteobacteria;o__Methylococcales;  f__Methylomonadaceae;g__Methylomonas;s__ |
| LL-enrich-bin28 | 547 | 3908447 | 365.183 | 98.04 | 7.89 | d__Bacteria;p__Proteobacteria;c__Gammaproteobacteria;o__Burkholderiales;  f__Methylophilaceae;g__Methylotenera |

**Table S3.** Composition of the sample that was used for metagenome sequencing.

|  | Metagenome sample |
| --- | --- |
| *Methylobacter spp.* | 22 |
| *Methylomonas spp.* | 17 |
| Other  Methylococcales | 0.3 |
| *Methylotenera spp.* | 24 |
|  |  |
| Total 16S rRNA copies per L^-1^ | 8.1 x10^5^ |
| Methanotroph 16S rRNA copies per L^-1^ | 3.2 x10^5^ |

**Table S4.** Relative abundance (%) of *Methylobacter* OTUs (>0.4% in at least one of the samples) in respect to the total 16S rRNA gene reads in the amplicon sequencing analysis for each incubation and for the enrichment sample used for the metagenomic sequencing.

|  |  |  | “Electron acceptor experiment” | | | | | | “O_2_-concentration experiment” | | | | Enrichment |
| --- | --- | --- | --- | --- | --- | --- | --- | --- | --- | --- | --- | --- | --- |
|  | Starting sample | Enrichment culture | Control, oxic | Nitrate, oxic | Control, anoxic | Nitrate, anoxic | Sulfate, anoxic | Humics, anoxic | Satu-rated | Micro-oxic | Trace | Anoxic | Metagenome |
| LLE-16S-1 | 0.0 | 0.1 | 1.6 | 0.3 | 0.0 | 0.1 | 0.1 | 0.0 | 0.3 | 0.1 | 0.3 | 0.1 | 0.8 |
| LLE-16S-2 | 0.0 | 0.7 | 1.3 | 1.1 | 1.0 | 0.2 | 0.2 | 0.0 | 0.8 | 0.3 | 1.1 | 0.1 | 0.0 |
| LLE-16S-3 | 0.0 | 0.0 | 0.6 | 0.0 | 0.0 | 0.4 | 0.2 | 0.0 | 0.2 | 0.0 | 0.0 | 0.3 | 0.0 |
| LLE-16S-4 | 0.0 | 0.3 | 1.0 | 0.2 | 0.0 | 0.3 | 0.1 | 0.0 | 0.1 | 0.1 | 0.0 | 0.1 | 0.6 |
| LLE-16S-5 | 0.0 | 0.3 | 0.8 | 0.3 | 0.0 | 0.2 | 0.2 | 0.0 | 0.1 | 0.1 | 0.2 | 0.2 | 0.4 |
| LLE-16S-6 | 0.0 | 0.7 | 0.7 | 0.1 | 0.0 | 0.2 | 0.0 | 0.0 | 0.1 | 0.1 | 0.0 | 0.2 | 0.1 |
| LLE-16S-7 | 0.0 | 10 | 0.5 | 1.4 | 0.0 | 0.9 | 0.2 | 0.0 | 0.2 | 0.2 | 0.5 | 0.6 | 1.3 |
| LLE-16S-8 | 0.0 | 0.7 | 0.5 | 0.1 | 0.2 | 0.4 | 0.5 | 0.0 | 0.2 | 0.5 | 0.4 | 0.4 | 0.0 |
| LLE-16S-9 | 0.0 | 0.3 | 0.3 | 0.1 | 0.5 | 0.5 | 0.3 | 0.0 | 0.1 | 0.3 | 0.0 | 0.5 | 0.0 |
| LLE-16S-10 | 0.0 | 0.7 | 0.6 | 0.2 | 0.0 | 0.2 | 0.1 | 0.0 | 0.2 | 0.1 | 0.1 | 0.3 | 0.2 |
| LLE-16S-11 | 0.0 | 0.1 | 1.2 | 0.3 | 0.0 | 0.1 | 0.1 | 0.0 | 0.1 | 0.0 | 0.0 | 0.1 | 0.0 |
| LLE-16S-12 | 0.0 | 5.0 | 0.6 | 1.1 | 0.0 | 0.6 | 0.3 | 0.0 | 0.3 | 0.2 | 0.3 | 0.5 | 0.9 |
| LLE-16S-13 | 0.0 | 0.3 | 0.5 | 0.5 | 0.0 | 0.1 | 0.1 | 0.0 | 0.1 | 0.1 | 0.1 | 0.1 | 0.1 |
| LLE-16S-14 | 0.0 | 0.0 | 0.6 | 0.6 | 0.0 | 0.2 | 0.1 | 0.0 | 0.1 | 0.1 | 0.2 | 0.1 | 0.5 |
| LLE-16S-15 | 0.0 | 0.1 | 0.3 | 0.1 | 0.1 | 0.2 | 0.3 | 0.0 | 0.2 | 0.3 | 0.1 | 0.7 | 0.0 |
| LLE-16S-16 | 0.0 | 0.0 | 0.5 | 0.1 | 0.0 | 0.2 | 0.2 | 0.0 | 0.2 | 0.2 | 0.1 | 0.3 | 0.2 |
| LLE-16S-17 | 0.0 | 0.2 | 0.2 | 0.0 | 0.1 | 0.2 | 0.1 | 0.0 | 0.4 | 0.2 | 0.2 | 0.5 | 0.0 |
| LLE-16S-18 | 0.0 | 0.1 | 0.3 | 0.7 | 0.1 | 0.1 | 0.0 | 0.0 | 0.4 | 0.2 | 0.2 | 0.4 | 0.0 |
| LLE-16S-19 | 0.0 | 0.1 | 0.5 | 1.1 | 0.0 | 0.4 | 0.2 | 0.0 | 0.3 | 0.2 | 0.0 | 0.5 | 0.3 |
| LLE-16S-20 | 0.0 | 0.1 | 0.1 | 0.4 | 0.0 | 0.1 | 0.0 | 0.0 | 0.0 | 0.0 | 0.0 | 0.1 | 0.0 |
| LLE-16S-21 | 0.0 | 0.4 | 0.1 | 0.3 | 0.1 | 0.1 | 0.4 | 0.0 | 0.0 | 0.1 | 0.1 | 0.2 | 0.0 |
| LLE-16S-22 | 0.0 | 0.4 | 0.5 | 0.8 | 0.0 | 0.2 | 0.1 | 0.0 | 0.0 | 0.1 | 0.1 | 0.1 | 0.2 |
| LLE-16S-23 | 0.0 | 0.0 | 0.5 | 1.4 | 0.0 | 0.0 | 0.0 | 0.0 | 0.0 | 0.4 | 0.3 | 0.3 | 0.3 |
| LLE-16S-24 | 0.0 | 0.1 | 0.2 | 0.4 | 0.0 | 0.3 | 0.1 | 0.0 | 0.1 | 0.1 | 0.1 | 0.1 | 0.2 |
| LLE-16S-25 | 0.0 | 0.0 | 0.1 | 0.4 | 0.1 | 0.2 | 0.1 | 0.0 | 0.0 | 0.6 | 0.2 | 0.4 | 0.0 |
| LLE-16S-26 | 0.0 | 0.0 | 0.1 | 0.5 | 0.0 | 0.0 | 0.0 | 0.0 | 0.1 | 0.0 | 0.0 | 0.1 | 0.0 |
| LLE-16S-27 | 0.0 | 0.0 | 0.0 | 0.5 | 0.0 | 0.0 | 0.0 | 0.0 | 0.1 | 0.0 | 0.0 | 0.0 | 0.0 |

**Table S5.** P-value of t-tests between *Methylobacter* relative abundances of the three replicates per incubation type, indicating whether the difference in relative abundance between sample categories was statistically significant.

| *Methylobacter* |  |  | | |  |  |  |  |  |  |  |
| --- | --- | --- | --- | --- | --- | --- | --- | --- | --- | --- | --- |
|  | Oxic control | Oxic nitrate | Anoxic control | Anoxic nitrate | Anoxic sulfate |  |  |  |  |  |  |
| Oxic control |  | 0.13 | 2.2E-05 | 0.002 | 0.0002 |  |  |  |  |  |  |
| Oxic nitrate |  |  | 0.001 | 0.02 | 0.004 |  |  |  |  |  |  |
| Anoxic control |  |  |  | 0.01 | 0.02 |  |  |  |  |  |  |
| Anoxic nitrate |  |  |  |  | 0.08 |  |  |  |  |  |  |
| Anoxic sulfate |  |  |  |  |  |  |  |  |  |  |  |
|  |  |  |  |  |  |  |  |  |  |  |  |
